# EPIGENETIC AGE DYNAMICS AND EMBRYONIC MORTALITY POINT TO GASTRULATION AS AGING GROUND ZERO IN XENOPUS LAEVIS

**DOI:** 10.1093/geroni/igad104.2485

**Published:** 2023-12-21

**Authors:** Bohan Zhang, Vadim Gladyshev

**Affiliations:** Brigham and Women’s Hospital, Boston, Massachusetts, United States; Harvard Medical School, Boston, Massachusetts, United States

## Abstract

Recent studies on embryonic development revealed a natural rejuvenation event followed by epigenetic aging. Here, we developed a frog aging clock based on the DNA methylation profile in the African clawed frog Xenopus laevis. Application of this clock to Xenopus laevis early embryonic development identified a decrease in epigenetic age during early embryogenesis, with the minimal epigenetic age reached around gastrulation. We further examined the individual developmental trajectories of 6,457 embryos and found that this minimum age is also accompanied by a higher incidence of disrupted development. Taken together, our data point to gastrulation as a critical stage for aging and natural rejuvenation, characterized by the lowest epigenetic age and increased mortality, defining aging ground zero as the basepoint where rejuvenation ends and the aging process begins.

